# Metabolomics and Incidence of Atrial Fibrillation in African Americans: The Atherosclerosis Risk in Communities (ARIC) Study

**DOI:** 10.1371/journal.pone.0142610

**Published:** 2015-11-06

**Authors:** Alvaro Alonso, Bing Yu, Waqas T. Qureshi, Morgan E. Grams, Elizabeth Selvin, Elsayed Z. Soliman, Laura R. Loehr, Lin Y. Chen, Sunil K. Agarwal, Danny Alexander, Eric Boerwinkle

**Affiliations:** 1 Division of Epidemiology and Community Health, School of Public Health, University of Minnesota, Minneapolis, Minnesota, United States of America; 2 Human Genetics Center, University of Texas Health Science Center at Houston, Houston, Texas, United States of America; 3 Division of Cardiovascular Medicine, Wake Forest University School of Medicine, Winston-Salem, North Carolina, United States of America; 4 Department of Medicine, Johns Hopkins University School of Medicine, Baltimore, Maryland, United States of America; 5 Department of Epidemiology, Johns Hopkins Bloomberg School of Public Health, Baltimore, Maryland, United States of America; 6 Welch Center for Prevention, Epidemiology and Clinical Research, Johns Hopkins University, Baltimore, Maryland, United States of America; 7 Epidemiological Cardiology Research Center (EPICARE), Department of Epidemiology and Prevention, Wake Forest School of Medicine, Winston-Salem, North Carolina, United States of America; 8 Department of Medicine-Cardiology, Wake Forest School of Medicine, Winston-Salem, North Carolina, United States of America; 9 Department of Epidemiology, Gillings School of Global Public Health, University of North Carolina, Chapel Hill, North Carolina, United States of America; 10 Cardiovascular Division, Department of Medicine, University of Minnesota Medical School, Minneapolis, Minnesota, United States of America; 11 Mount Sinai Heart Hospital, New York, New York, United States of America; 12 Metabolon, Inc., Durham, North Carolina, United States of America; 13 Human Genome Sequencing Center, Baylor College of Medicine, Houston, Texas, United States of America; Medical University Hamburg, University Heart Center, GERMANY

## Abstract

**Background:**

Atrial fibrillation (AF) is a common arrhythmia. Application of metabolomic approaches, which may identify novel pathways and biomarkers of disease risk, to a longitudinal epidemiologic study of AF has been limited.

**Methods:**

We determined the prospective association of 118 serum metabolites identified through untargeted metabolomics profiling with the incidence of newly-diagnosed AF in 1919 African-American men and women from the Atherosclerosis Risk in Communities study without AF at baseline (1987–1989). Incident AF cases through 2011 were ascertained from study electrocardiograms, hospital discharge codes, and death certificates.

**Results:**

During a median follow-up of 22 years, we identified 183 incident AF cases. In Cox proportional hazards models adjusted for age, sex, smoking, body mass index, systolic blood pressure, use of antihypertensive medication, diabetes, prevalent heart failure, prevalent coronary heart disease, and kidney function, two conjugated bile acids (glycolithocholate sulfate and glycocholenate sulfate) were significantly associated with AF risk after correcting for multiple comparisons (p<0.0004). Multivariable-adjusted hazard ratios (95% confidence intervals) of AF were 1.22 (1.12–1.32) for glycolithocholate sulfate and 1.22 (1.10–1.35) for glycocholenate sulfate per 1-standard deviation higher levels. Associations were not appreciably different after additional adjustment for alcohol consumption or concentrations of circulating albumin and liver enzymes.

**Conclusion:**

We found an association of higher levels of two bile acids with an increased risk of AF, pointing to a potential novel pathway in AF pathogenesis. Replication of results in independent studies is warranted.

## Introduction

Atrial fibrillation (AF) is a common cardiac arrhythmia associated with increased mortality and an elevated risk of stroke, heart failure, myocardial infarction, and dementia.[1] The last two decades have seen major advances in our understanding of the pathophysiology of AF; however, key knowledge gaps remain.[2] Identification of novel biomarkers of AF risk could shed new light into relevant biological pathways and open new avenues for development of preventive and therapeutic strategies.[3] In this regard, large-scale, high-throughput “omics” techniques, such as genomics, epigenomics, transcriptomics, proteomics, and metabolomics, offer a unique opportunity to discover new mechanisms and to refine our comprehension of established etiopathogenic pathways.[4]

The application of metabolomics—the systematic study of small molecules in a particular tissue—to research on cardiac arrhythmias, specifically AF, has been limited. Published studies have examined the metabolomic profile of atrial tissue from AF patients or from a canine model of AF.[5, 6] These studies found changes in molecules involved in energy metabolism compared to AF-free controls. However, no prospective assessments of metabolomic profiling with risk of AF have been previously published. To address this gap, we explored the association of molecules identified through untargeted metabolomics with risk of newly-diagnosed AF in a subset of participants of the Atherosclerosis Risk in Communities (ARIC) study.

## Methods

### Study sample

The ARIC study is a prospective cohort originally designed to assess risk factors for cardiovascular disease in the general population. A total of 15,792 men and women age 45–64 years old were recruited from four communities (Forsyth County, North Carolina; Jackson, Mississippi; northwest suburbs of Minneapolis, Minnesota; and Washington County, Maryland) in 1987–89. Participants were mostly white in the Minneapolis and Washington County sites, white and African-American in Forsyth County, while only African-American individuals were recruited in Jackson. Study details have been published elsewhere.[7] After the baseline examination, participants were invited for four follow-up visits in 1990–92, 1993–95, 1996–98, and 2011–13. For the present analysis, we included 1919 African-American participants from the Jackson site examined at the baseline visit with metabolomics profiling and without evidence of AF at recruitment. The ARIC study has been approved by the Institutional Review Board at the University of Minnesota, Johns Hopkins University, Wake Forest University, University of North Carolina, University of Texas Health Sciences Center at Houston, and University of Mississippi Medical Center. Participants provided written informed consent.

### Metabolomic profiling

As previously described in detail in the context of a study assessing metabolomic predictors of heart failure [8], metabolomic profiles were performed in 2010 in serum samples obtained from a subset of 1977 African Americans in the Jackson field center. These samples had been kept at -80°C since their collection in 1987–89 and were assayed with an untargeted, gas chromatography/mass spectrometry and liquid chromatography/mass spectrometry–based metabolomic quantification protocol by Metabolon, Inc. (Durham, North Carolina). This approach identified and quantified named compounds with known chemical identities as well as unnamed compounds without current chemical standards. For the present analysis, we considered 118 named compounds with adequate medium-term reliability (defined as a reliability coefficient ≥0.6 in repeat samples obtained 4–6 weeks apart in 60 individuals)[9] and ≤80% of missing values or below the limit of detection.

### Ascertainment of AF

A thorough description of AF ascertainment in the ARIC study has been previously published.[10, 11] Briefly, cases of AF were identified from three sources: electrocardiograms (ECG) at the study examinations, hospital discharge codes, and death certificates. At all examinations, participants underwent a standard 12-lead ECG with MAC PC ECG machines (Marquette Electronics, Milwaukee, WI). Digital ECG information was transferred to the ARIC ECG Reading Center located at the Epidemiological Cardiology Research Center, Wake Forest School of Medicine, Winston Salem, NC, where it was automatically processed using GE Marquette 12-SL program (GE Marquette, Milwaukee, WI). All automatically detected AF cases were reviewed by an experienced cardiologist. Hospitalizations during follow-up are identified through annual follow-up calls (response rate >90%) and surveillance of local hospitals, and hospitalization discharge codes are recorded. AF was considered present if ICD-9-CM codes 427.31 or 427.32 were present in a hospitalization in any position not accompanied by a procedure code for open cardiac surgery. This approach for case ascertainment has demonstrated adequate validity in the ARIC cohort and other studies.[10, 12] Finally, AF was considered present if the death certificate included ICD-9 code 427.3 or ICD-10 code I48.

### Assessment of other covariates

At baseline, information on age, sex, race, and smoking status was self-reported. Alcohol consumption was ascertained by an interviewer-administered questionnaire. Height and weight were measured with the participant lightly dressed. Body mass index was calculated as weight in kilograms divided by height in meters squared. Sitting blood pressure was measured three times using a random-zero sphygmomanometer after five minutes of rest, and the second and third measurements were averaged. Diabetes was defined as a fasting blood glucose ≥126 mg/dL, non–fasting blood glucose ≥200 mg/dL, a self-reported physician diagnosis of diabetes, or current use of antidiabetic medication. Estimated glomerular filtration rate (eGFR_CKD-EPI_) was calculated from serum creatinine using the CKD-EPI equation.[13] Serum albumin was measured with a Coulter DACOS (Coulter Diagnostics) using Coulter’s bromcresol green colorimetric assay. Liver enzymes (aspartate aminotransferase, alanine aminotransferase, gamma-glutamyl transpeptidase) were measured in serum samples collected at visit 2 (1990–1992) using Roche reagents on the Roche Modular P800 Chemistry analyzer (Roche Diagnostics Corporation). Prevalent heart failure was defined using the Gothenburg criteria,[14] while prevalent coronary heart disease was considered present if the participant self-reported a history of myocardial infarction, coronary bypass surgery, or coronary angioplasty, or had evidence of a previous myocardial infarction by ECG at the baseline visit.

### Statistical analysis

The association of each metabolite with newly-diagnosed AF was evaluated using Cox proportional hazards regression. Time to follow-up was defined as the time between the baseline examination and the incidence of AF, death, loss to follow-up, or December 31, 2011, whichever occurred first. Metabolites with <50% missing or below the detection limit values were mean centered and modeled as continuous variables in standard deviation units; missing values for this group were imputed using the lowest detectable value in the study sample. Metabolites with 50–80% missing or below the detection limit values were modeled as an ordinal variable with 3 levels: missing/below the detection limit, detected below the median, and detected equal or above the median. Models were initially adjusted for age and sex. A second model additionally adjusted for smoking status (current vs former/never), body mass index, systolic blood pressure, hypertension medications, diabetes, prevalent heart failure, and prevalent coronary heart disease. Finally, a third model added eGFR_CKD-EPI_ to the previously listed covariates. Significance tests were corrected using the Dubey/Armitage-Parmar algorithm, a modified Bonferroni procedure.[15] Applying this method to the metabolomics data, two-tailed p-values <0.0004 were considered statistically significant. Because the two metabolites significantly associated with AF risk in our analysis may be considered markers of liver function or damage (see below), we conducted a sensitivity analysis further adjusting for baseline serum albumin, baseline alcohol consumption (in grams/week), and liver enzymes (aspartate aminotransferase, alanine aminotransferase, gamma-glutamyl transpeptidase) measured at visit 2. Also, given the role of bile acids in cholesterol metabolism and the potential link of blood lipids with AF incidence [16, 17], we conducted an analysis adjusting for baseline serum LDL cholesterol, HDL cholesterol and triglycerides. Additionally, we performed sex-specific analysis for the two metabolites significantly associated with AF risk.

The proportional hazards assumption was assessed with Schoenfeld residuals and including interaction terms between time and the corresponding metabolite in the regression model. We explored the shape of the association between the significant metabolites and AF risk by modeling the metabolite as restricted cubic splines with knots at the 5^th^, 27.5^th^, 50^th^, 72.5^th^, and 95^th^ percentiles, as recommended by Harrell.[18] Finally, to determine the joint association of the two significant metabolites with AF risk, we conducted two additional analyses. First, we ran a multivariable Cox model including both metabolites simultaneously. Second, we categorized the study participants by quintiles of the two metabolites and created a new variable as the sum of the quintiles rank (range 2–10); this variable was modeled both as a continuous and a categorical variable (with individuals in the bottom quintile of both metabolites as the reference group).

All analyses were conducted using SAS version 9.2 or 9.3 (SAS Institute, Inc., Cary, NC).

## Results

Of 1977 participants with available metabolomics profiling, 58 were excluded because they had prevalent AF or did not have a baseline electrocardiogram, leaving 1919 eligible individuals. During a median follow-up of 22 years, 183 incident cases of AF were identified (incidence rate: 5.0 per 1000 person-years). Table 1 reports selected baseline characteristics by AF status during follow-up. Compared to those without diagnosed AF during follow-up, those who developed AF were slightly older, more likely to be male and current smokers, had higher body mass index and systolic blood pressure and higher prevalence of diabetes and prevalent cardiovascular diseases.

**Table 1:** Selected baseline characteristics by atrial fibrillation (AF) status during follow-up in a subsample (N = 1919) of participants from the Atherosclerosis Risk in Communities (ARIC) study Jackson, Mississippi field center without AF at baseline (1987–1989).

Baseline characteristics*	No incident AF	Incident AF
N	1736	183
Age, years	53 (6)	56 (6)
Women, %	65.4	56.8
Body mass index, kg/m^2^	29.5 (6.0)	30.9 (6.2)
Current smokers, %	28.1	35.0
Systolic blood pressure, mmHg	127 (21)	137 (24)
Anti-hypertensive medication, %	36.3	51.4
Diabetes, %	14.6	29.5
eGFR_CKD-EPI_, mL/min/1.73 m^2^	113 (18)	109 (20)
Prevalent heart failure, %	4.4	8.7
Prevalent coronary heart disease, %	3.2	8.7

*Values correspond to means (standard deviation) or percentages

eGFR_CKD-EPI_: estimated glomerular filtration rate

**Fig 1:**
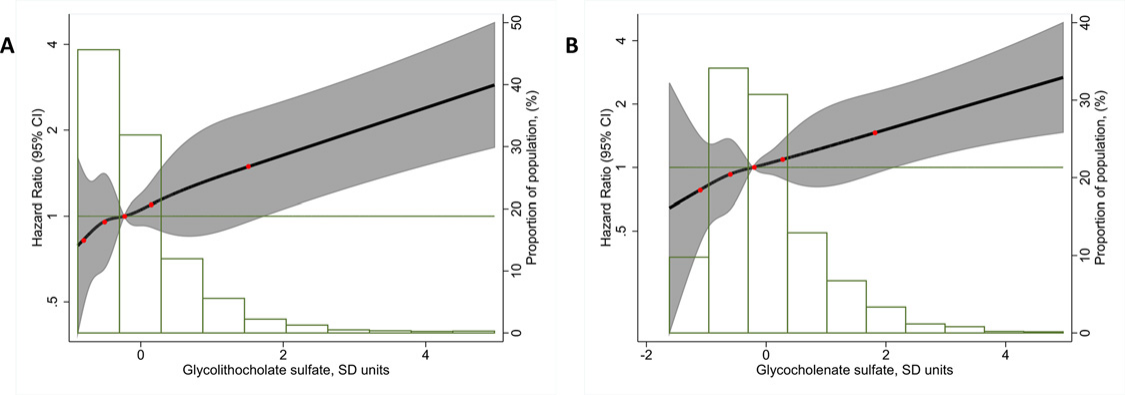
Association of concentrations of glycolithocholate sulfate (A, left panel) and glycocholenate sulfate (B, right panel) with incidence of atrial fibrillation presented as hazard ratio (HR; solid line) and 95% confidence intervals (CI; shaded area). Results from Cox proportional hazards model with metabolites modeled using restricted cubic splines (knots at 5^th^, 27.5^th^, 50^th^, 72.5^th^, and 95^th^ percentiles), adjusted for age, sex, body mass index, smoking, diabetes, systolic blood pressure, use of antihypertensive medication, prevalent coronary heart disease, prevalent heart failure, and eGFR_CKD-EPI_. Median value of the metabolite was considered the reference (HR = 1). The histograms represent the frequency distribution of both metabolites in the study sample. The red dots indicate the position of the knots used in the restricted cubic splines. Atherosclerosis Risk in Communities Study subsample, 1990–2011. eGFR_CKD-EPI_: CKD-EPI creatinine-based estimated glomerular filtration rate.

In models adjusted for age and sex, 8 metabolites were significantly associated with AF risk (p<0.0004) (Table 2). After additional adjustment for several AF risk factors, two metabolites, the bile acids glycolithocholate sulfate and glycocholenate sulfate, remained significantly associated. The hazard ratios (HR) and 95% confidence interval (CI) of AF per 1-standard deviation higher levels were 1.22, 95%CI 1.12–1.32 for glycolithocholate sulfate, and 1.22, 95%CI 1.10–1.35 for glycocholenate sulfate. Further adjustment for kidney function did not change the associations (Table 2). When modeled as restricted cubic splines, both glycolithocholate sulfate and glycocholenate sulfate showed roughly linear associations with the HR for AF (Fig 1). Complete results for the 118 metabolites are provided as a supplementary file (S1 Table). We did not find any evidence of violation of the proportional hazards assumption.

**Table 2:** Association of individual metabolites with incidence of atrial fibrillation, ARIC subsample, 1987–2011. Only metabolites that were statistically significant at p<0.0004 in the age- and sex-adjusted model are shown.

Metabolite	Model 1		Model 2		Model 3	
	HR (95%CI)	P-value	HR (95%CI)	P-value	HR (95%CI)	P-value
Glycolithocholate sulfate	1.23 (1.15, 1.32)	7.4×10^−9^	1.22 (1.12, 1.32)	1.8×10^−6^	1.22 (1.13, 1.32)	1.2×10^−6^
Glycocholenate sulfate	1.29 (1.18, 1.40)	1.3×10^−8^	1.22 (1.10, 1.35)	0.0002	1.22 (1.10, 1.36)	0.0001
Erythritol	1.32 (1.19, 1.47)	2.1×10^−7^	1.22 (1.08, 1.38)	0.002	1.23 (1.06, 1.42)	0.005
Hexanoylcarnitine	1.19 (1.08, 1.30)	0.0003	1.14 (1.03, 1.27)	0.01	1.14 (1.03, 1.27)	0.01
Mannose	1.32 (1.21, 1.45)	2.3×10^−9^	1.19 (1.03, 1.37)	0.02	1.20 (1.04, 1.38)	0.01
Glucose	1.37 (1.23, 1.52)	4.0×10^−9^	1.19 (1.03, 1.39)	0.02	1.20 (1.03, 1.39)	0.02
o-cresol sulfate	1.23 (1.10, 1.38)	0.0003	1.11 (0.96, 1.28)	0.17	1.09 (0.95, 1.27)	0.22
Cotinine	1.38 (1.16, 1.65)	0.0004	1.08 (0.83, 1.41)	0.56	1.08 (0.83, 1.41)	0.57

Model 1: Cox proportional hazards model adjusted for age and sex. Model 2: As Model 1, additionally adjusted for smoking, body mass index, systolic blood pressure, use of antihypertensive medications, diabetes mellitus, prevalent heart failure, and prevalent coronary heart disease. Model 3: As Model 2, additionally adjusted for eGFR_CKD-EPI_

We performed some additional analyses. Since sulfated bile acids have been described as potential markers of hepatobiliary diseases, we performed an analysis adjusting for baseline serum albumin, alcohol consumption, and liver enzymes measured at visit 2. After these additional adjustments, both glycolithocholate sulfate and glycocholenate sulfate remained associated with AF incidence (HR, 95%CI: 1.18, 1.09–1.29 and 1.23, 1.10–1.36, respectively). Likewise, both metabolites showed similar associations with AF incidence after adjustment for baseline serum LDL cholesterol, HDL cholesterol and triglycerides (HR, 95%CI: 1.17, 1.08–1.27 for glycolithocholate sulfate and 1.18, 1.07–1.30 for glycocholenate sulfate). We also explored the association of both metabolites with incidence of AF in men and women separately. No differences were observed in the association of glycocholenate sulfate with AF incidence by sex; however, glycolithocolate sulfate was more strongly associated with AF incidence in men than in women (p for interaction < 0.001) (S2 Table).

Finally, we assessed whether associations of both metabolites with AF risk were independent of each other. The two bile acids were moderately correlated (r = 0.45). When included simultaneously in a multivariable Cox model, the association of both metabolites with AF incidence became weaker and remained significant at the traditional p = 0.05 level only for glycolithocholate sulfate, but not for glycocholenate sulfate (HR 1.16, 95%CI 1.02–1.31, p = 0.02 for glycolithocholate sulfate and 1.09, 95%CI 0.94–1.25, p = 0.25 for glycocholenate sulfate). When the circulating levels of both metabolites were jointly modeled as the sum of their quintiles (rank 2 to 10), the association became stronger: the HR (95%CI) of AF among participants in the top quintile of both bile acids, compared to those in the bottom quintiles, was 2.99 (1.12–7.99), and the p-value for the sum of quintiles modeled as a continuous variable was 0.003.

## Discussion

In this systematic assessment of circulating metabolites and AF risk, we found that increased levels of two conjugated bile acids, glycolithocholate sulfate and glycocholenate sulfate, were associated with increased incidence of AF. This association was independent of other risk factors for AF, including kidney function, and of alcohol consumption and markers of liver damage and function.

To date, the use of metabolomics in the study of AF has been limited to cross-sectional studies. Mayr and colleagues used a combined metabolomic and proteomic approach in human atrial tissue from patients with AF and controls in sinus rhythm [5]. Using high-resolution proton nuclear magnetic resonance spectroscopy, the authors found that levels of beta-hydroxybutyrate, involved in ketone body metabolism, ketogenic amino acids, and glycine were elevated in cardiac tissue from patients with persistent AF. Also employing a combined metabolomics and proteomic approach, De Souza and colleagues studied left-atrial cardiomyocytes of ventricular-tachypaced dogs, an animal model of heart failure-induced AF, and sham controls [6]. Observed metabolic changes suggested increased metabolic stress paired with inefficient energy utilization, and a shift from glycolysis to ketoacid metabolism. In contrast to our analysis of the ARIC cohort, these two studies focused on metabolomics of AF-affected cardiac tissue, not directly addressing the association of circulating metabolites with the risk of developing AF.

The two molecules associated with AF risk in our study are glycine-conjugated bile acids that have also undergone sulfation. Specifically, glycolithocholate sulfate is derived from glycine-conjugated lithocholic acid, a secondary bile acid synthesized by intestinal bacteria from chenodeoxycholic acid, a primary bile acid excreted by the liver. Lithocholic acid in abnormally high concentrations can be cytotoxic; sulfation reduces its toxic effects through increased hydrophilicity and enhanced fecal and urinary excretion [19]. High levels of glycolithocolate sulfate in urine have been associated with presence of chronic hepatic disease, suggesting a potential role as marker of liver damage [20]. Glycocholenate sulfate, on the other hand, is possibly synthesized from glycine-amidation and sulfation of 3-beta-hydroxy-5-cholenoic acid (also known as cholenate). Previous literature has described elevations of 3-beta-hydroxy-5-cholenoic acid in patients with liver disease, particularly in those with primary biliary cirrhosis [21], a condition characterized by progressive inflammatory destruction of the bile ducts, leading to cirrhosis. The untargeted metabolomics assessment measured other bile acids in serum, including glycocholate, glycodeoxycholate, and ursodeoxycholate, but none of these were significantly associated with AF risk after correction for multiple comparisons.

The mechanism responsible for the association of higher levels of bile acids with risk of AF is unclear. Both glycolithocolate sulfate and glycocholenate sulfate may be elevated in the context of liver disease. We and others have shown that higher circulating levels of liver enzymes, markers of liver damage, are associated with an increased risk of AF [22, 23]. In the present analysis, however, the association of the two bile acids with higher AF risk was independent of markers of liver damage and function, pointing to different pathways underlying this association. Limited previous evidence has linked bile acids with the occurrence of arrhythmias. Experiments in rat ventricular muscle have demonstrated negative inotropic effects of high levels of bile acids [24], and studies in neonatal rat cardiomyocyte cultures have also demonstrated that taurine-conjugated bile acids cause bradycardia and loss of synchronous beating [25]. Moreover, intrahepatic cholestasis of pregnancy, a disorder characterized by elevated maternal serum bile acids, has been linked to presence of fetal cardiac arrhythmias [26]. Recently, Rainer and colleagues explored the potential arrhythmogenic effects of bile acids in the adult human heart [27]. Their studies found that increasing concentrations of taurine- and glycine-conjugated bile acids caused increased occurrence of arrhythmic extra contractions in myocardial tissue extracted from the right atrium of adult patients undergoing heart surgery. They also found higher concentrations of non-ursodeoxycholate bile acids in patients with AF compared with controls in sinus rhythm [27]. These findings, together with growing evidence of the systemic metabolic effects of circulating bile acids through activation of farnesoid X receptor [28], and the effect that the gut microbiota (responsible for secondary bile acids synthesis) has in cardiometabolic health [29], suggest that future research should pay additional attention to the role of bile acids in cardiac arrhythmogenesis.

Strengths of our study include the well-characterized cohort with excellent follow-up and availability of rigorous information on potential confounders. Some limitations need to be mentioned. First, our method of AF ascertainment probably missed asymptomatic cases and those managed exclusively in the outpatient setting. Previous work, however, has shown the high specificity and validity of AF ascertainment using hospitalization discharge codes in the ARIC cohort and other epidemiologic studies [10, 12]. Second, we only had single metabolomic assessments and this may be insufficient to characterize long-term levels of these metabolites. Nonetheless, as described in the methods, we only considered in our analysis metabolites with adequate medium-term reliability [9]. Third, we do not have direct evidence of the stability of the metabolite profile over the more than 20 years that the samples remained in storage. Finally, an additional limitation includes the unavailability of a replication sample. Although not a limitation, restriction of our study to African Americans makes uncertain the generalizability of our results to other racial groups.

## Conclusions

We have identified a novel association of two secondary bile acids with the incidence of AF. Replication of these results in additional studies is critical. If our observations are replicated, additional research should address the potential pathways linking bile acid metabolism with the etiopathogenesis of AF.

## Supporting Information

S1 Table Complete results for the association of 118 metabolites with incidence of atrial fibrillation, ARIC subsample, 1987–2011. (XLSX)

S2 Table Association of glycolithocholate sulfate and glycocholenate sulfate with incidence of atrial fibrillation by sex, ARIC subsample, 1987–2011. (DOCX)

## References

[1] Mozaffarian D, Benjamin EJ, Go AS, Arnett DK, Blaha MJ, Cushman M, et al Heart disease and stroke statistics-2015 update: a report from the American Heart Association. Circulation. 2015;131:e29–e322. 10.1161/CIR.0000000000000152 25520374

[2] Van Wagoner DR, Piccini JP, Albert CM, Anderson ME, Benjamin EJ, Brundel B, et al Progress toward the prevention and treatment of atrial fibrillation: A summary of the Heart Rhythm Society Research Forum on the Treatment and Prevention of Atrial Fibrillation, Washington, DC, December 9–10, 2013. Heart Rhythm. 2015;12:e5–e29. 10.1016/j.hrthm.2014.11.011 25460864 PMC4425127

[3] Rienstra M, McManus DD, Benjamin EJ. Novel risk factors for atrial fibrillation: useful for risk prediction and clinical decision making? Circulation. 2012;125:e941–e6. 10.1161/CIRCULATIONAHA.112.112920 22615425 PMC3725394

[4] Roberts LD, Gerszten RE. Toward new biomarkers of cardiometabolic diseases. Cell Metab. 2013;18:43–50. 10.1016/j.cmet.2013.05.009. 10.1016/j.cmet.2013.05.009 23770128 PMC3960498

[5] Mayr M, Yusuf S, Weir G, Chung Y-L, Mayr U, Yin X, et al Combined metabolomic and proteomic analysis of human atrial fibrillation. J Am Coll Cardiol. 2008;51:585–94. 10.1016/j.jacc.2007.09.055. 10.1016/j.jacc.2007.09.055 18237690

[6] De Souza AI, Cardin S, Wait R, Chung Y-L, Vijayakumar M, Maguy A, et al Proteomic and metabolomic analysis of atrial profibrillatory remodelling in congestive heart failure. J Mol Cell Cardiol. 2010;49:851–63. 10.1016/j.yjmcc.2010.07.008. 10.1016/j.yjmcc.2010.07.008 20655923

[7] The ARIC Investigators. The Atherosclerosis Risk in Communities (ARIC) study: design and objectives. Am J Epidemiol. 1989;129:687–702. 2646917

[8] Zheng Y, Yu B, Alexander D, Manolio TA, Aguilar D, Coresh J, et al Associations between metabolomic compounds and incident heart failure among African Americans: the ARIC Study. Am J Epidemiol. 2013;178:534–42. 10.1093/aje/kwt004 23788672 PMC3736751

[9] Zheng Y, Yu B, Alexander D, Couper DJ, Boerwinkle E. Medium-term variability of the human serum metabolome in the Atherosclerosis Risk in Communities (ARIC) study. OMICS. 2014;18:364–73. 10.1089/omi.2014.0019 24910946 PMC4048570

[10] Alonso A, Agarwal SK, Soliman EZ, Ambrose M, Chamberlain AM, Prineas RJ, et al Incidence of atrial fibrillation in whites and African-Americans: the Atherosclerosis Risk in Communities (ARIC) study. Am Heart J. 2009;158:111–7. 10.1016/j.ahj.2009.05.010 19540400 PMC2720573

[11] Soliman EZ, Prineas RJ, Case D, Zhang Z-M, Goff DC Jr. Ethnic distribution of electrocardiographic predictors of atrial fibrillation and its impact on understanding the ethnic distribution of ischemic stroke in the Atherosclerosis Risk in Communities Study (ARIC). Stroke. 2009;40:1204–11. 10.1161/STROKEAHA.108.534735 19213946 PMC2685189

[12] Jensen PN, Johnson K, Floyd J, Heckbert SR, Carnahan R, Dublin S. A systematic review of validated methods for identifying atrial fibrillation using administrative data. Pharmacoepidemiol Drug Saf. 2012;21 Suppl 1:141–7. 22262600 10.1002/pds.2317 PMC3674852

[13] Inker LA, Schmid CH, Tighiouart H, Eckfeldt JH, Feldman HI, Greene T, et al Estimating glomerular filtration rate from serum creatinine and cystatin C. N Engl J Med. 2012;367:20–9. 10.1056/NEJMoa1114248 . 22762315 PMC4398023

[14] Loehr LR, Rosamond WD, Chang PP, Folsom AR, Chambless LE. Heart failure incidence and survival (from the Atherosclerosis Risk in Communities Study). Am J Cardiol. 2008;101:1016–22. 10.1016/j.amjcard.2007.11.061 18359324

[15] Sankoh AJ, Huque MF, Dubey SD. Some comments on frequently used multiple endpoint adjustment methods in clinical trials. Stat Med. 1997;16:2529–42. 9403954 10.1002/(sici)1097-0258(19971130)16:22<2529::aid-sim692>3.0.co;2-j

[16] Lopez FL, Agarwal SK, MacLehose RF, Soliman EZ, Sharrett AR, Huxley RR, et al Blood lipid levels, lipid-lowering medications, and the incidence of atrial fibrillation: the Atherosclerosis Risk in Communities Study. Circ Arrhythm Electrophysiol. 2012;5:155–62. 10.1161/CIRCEP.111.966804 22227953 PMC3290134

[17] Alonso A, Yin X, Roetker NS, Magnani JW, Kronmal RA, Ellinor PT, et al Blood lipids and the incidence of atrial fibrillation: the Multi-Ethnic Study of Atherosclerosis and the Framingham Heart Study. J Am Heart Assoc. 2014;3:e001211 10.1161/JAHA.114.001211 25292185 PMC4323837

[18] Harrell FE Jr. Regression modeling strategies: with applications to linear models, logistic regression, and survival analysis New York: Springer; 2010.

[19] Alnouti Y. Bile acid sulfation: a pathway to bile acid elimination and detoxification. Toxicol Sci. 2009;108:225–46. 10.1093/toxsci/kfn268 19131563

[20] Kobayashi N, Katsumata H, Uto Y, Goto J, Niwa T, Kobayashi K, et al A monoclonal antibody-based enzyme linked immunosorbent assay of glycolithocholic acid sulfate in human urine for liver function test. Steroids. 2002;67:827–33. 12231118 10.1016/s0039-128x(02)00036-3

[21] Minder EI, Karlaganis G, Paumgartner G. Radioimmunological determination of serum 3beta-hydroxy-5-cholenoic acid in normal subjects and patients with liver disease. J Lipid Res. 1979;20:986–93. 93138

[22] Sinner MF, Wang N, Fox CS, Fontes JD, Rienstra M, Magnani JW, et al Relation of circulating liver transaminase concentrations to risk of new-onset atrial fibrillation. Am J Cardiol. 2013;111:219–24. 10.1016/j.amjcard.2012.09.021 23127690 PMC3538882

[23] Alonso A, Misialek JR, Amiin MA, Hoogeveen RC, Chen LY, Agarwal SK, et al Circulating levels of liver enzymes and incidence of atrial fibrillation: the Atherosclerosis Risk in Communities cohort. Heart. 2014;100:1511–6. 10.1136/heartjnl-2014-305756 24924619 PMC4225783

[24] Binah O, Rubinstein I, Bomzon A, Better OS. Effects of bile acids on ventricular muscle contraction and electrophysiological properties: studies in rat papillary muscle and isolated ventricular myocytes. Naunyn Schmiedebergs Arch Pharmacol. 1987;335:160–5. 3561530 10.1007/BF00177718

[25] Gorelik J, Shevchuk A, de Swiet M, Lab M, Korchev Y, Williamson C. Comparison of the arrhythmogenic effects of tauro- and glycoconjugates of cholic acid in an in vitro study of rat cardiomyocytes. BJOG. 2004;111:867–70. 15270939 10.1111/j.1471-0528.2004.00166.x

[26] Al Inizi S, Gupta R, Gale A. Fetal tachyarrhythmia with atrial flutter in obstetric cholestasis. Int J Gyneacol Obstet. 2006;93:53–4. 10.1016/j.ijgo.2005.12.030 16527280

[27] Rainer PP, Primessnig U, Harenkamp S, Doleschal B, Wallner M, Fauler G, et al Bile acids induce arrhythmias in human atrial myocardium—implications for altered serum bile acid composition in patients with atrial fibrillation. Heart. 2013;99:1685–92. 10.1136/heartjnl-2013-304163 23894089

[28] Schaap FG, Trauner M, Jansen PLM. Bile acid receptors as targets for drug development. Nat Rev Gastroenterol Hepatol. 2014;11:55–67. 10.1038/nrgastro.2013.151 23982684

[29] Le Chatelier E, Nielsen T, Qin J, Prifti E, Hildebrand F, Falony G, et al Richness of human gut microbiome correlates with metabolic markers. Nature. 2013;500:541–6. 10.1038/nature12506 Available: http://www.nature.com/nature/journal/v500/n7464/abs/nature12506.html#supplementary-information. 23985870

