# Supplementary material for: Metabolomics and Incidence of Atrial Fibrillation in African Americans: The Atherosclerosis Risk in Communities (ARIC) Study
Source: PLoS One. 2015 Nov 6;10(11):e0142610. doi: 10.1371/journal.pone.0142610 (PMC4636390; doi:10.1371/journal.pone.0142610)
Supplement: S2 Table — (DOCX) [file pone.0142610.s002.docx]

S2 Table. Association of glycolithocholate sulfate and glycocholenate sulfate with incidence of atrial fibrillation by sex, ARIC subsample, 1987-2011.

|  | Women (N = 1240; 104 AF events) | | Men (N = 679; 79 AF events) | | P for interaction |
| --- | --- | --- | --- | --- | --- |
|  | HR (95%CI) | P-value | HR (95%CI) | P-value |  |
| Glycolithocholate sulfate | | | | | |
| Model 1 | 1.19 (1.08, 1.31) | 0.0003 | 1.51 (1.27, 1.80) | 3.8×10^-6^ | 0.02 |
| Model 2 | 1.16 (1.05, 1.28) | 0.003 | 1.66 (1.37, 2.02) | 3.1×10^-7^ | 0.001 |
| Model 3 | 1.16 (1.05, 1.28) | 0.003 | 1.67 (1.37, 2.03) | 2.8×10^-7^ | 0.001 |
| Glycocholenate sulfate | | | | | |
| Model 1 | 1.21 (1.06, 1.38) | 0.005 | 1.44 (1.24, 1.67) | 1.1×10^-6^ | 0.09 |
| Model 2 | 1.14 (0.98, 1.32) | 0.08 | 1.32 (1.11, 1.55) | 0.001 | 0.20 |
| Model 3 | 1.14 (0.98, 1.32) | 0.08 | 1.32 (1.11, 1.55) | 0.001 | 0.20 |
